# Global landscape, research hotspots, and evolutionary trends in childhood adversity and chronic disease research: a bibliometric analysis based on three databases

**DOI:** 10.3389/fpubh.2026.1862548

**Published:** 2026-08-25

**Authors:** Yixia Zhou, Liuyin Jin, Weili Jiang, Lindan Sheng, Yang Li, Jianjiang Fang, Ying Wang

**Affiliations:** 1 The Affiliated Lihuili Hospital of Ningbo University, Ningbo, Zhejiang, China; 2 Second People's Hospital of Lishui City, Lishui, Zhejiang, China

**Keywords:** adverse childhood experiences, bibliometric analysis, childhood adversity, chronic disease, evolutionary trends, research hotspots

## Abstract

**Background:**

Adverse childhood experiences (ACEs) are increasingly recognized as important life-course determinants of chronic disease risk. However, the overall knowledge structure, research hotspots, and developmental trends in this field remain insufficiently characterized. This study aimed to systematically map the global research landscape of childhood adversity and chronic diseases through bibliometric analysis.

**Methods:**

Literature related to childhood adversity and chronic diseases was retrieved from the Web of Science Core Collection (WoSCC), Scopus, and PubMed from database inception to December 31, 2025. After data conversion, merging, screening, and deduplication, bibliometric analyses were conducted to evaluate publication trends, contributions of countries, institutions, authors, and journals, collaboration networks, keyword co-occurrence, and citation impact using R, VOSviewer, and CiteSpace.

**Results:**

A total of 5,369 publications were included. Annual publication output showed a sustained upward trend, especially after 2010, indicating rapidly growing scholarly interest in this field. The United States dominated in both publication output and citation impact and showed strong international collaboration, while the United Kingdom, Canada, Germany, and Australia were also major contributors. The University of California System and Harvard University were central in the institutional collaboration network. Keyword analysis identified major hotspots including childhood adversity/child abuse, depression, obesity, stress, risk factors, and cardiovascular disease, with emerging attention to chronic pain, chronic fatigue, and intergenerational effects. Highly cited studies were largely centered on the ACE framework and its associations with major adult health risks, reflecting a stable knowledge base for the field.

**Conclusions:**

Research on childhood adversity and chronic diseases has developed into a mature and expanding interdisciplinary field with a clear international collaboration structure and an evolving thematic focus. The field is shifting from early association-based research toward mechanistic exploration and evidence integration. Future studies should strengthen the standardization of exposure and outcome definitions, expand longitudinal and causal evidence, and promote risk-stratified prevention and translational research.

## 1 Introduction

Childhood is a critical stage for the development of an individual's physiological, psychological, and social functioning, and early-life environments and experiences exert profound influences on lifelong health outcomes (1). Adverse childhood experiences (ACEs) refer to a range of negative experiences occurring during childhood that may have sustained detrimental effects on physical and mental health. These typically include emotional abuse, physical abuse, sexual abuse, emotional or physical neglect, domestic violence, as well as caregiver mental illness, substance abuse, or family disruption (2). Previous studies have shown that ACEs rarely occur in isolation; rather, they often cluster within the same family and social environment. Early exposure to such adversities may exert long-term and complex effects on subsequent health through multiple biopsychosocial mechanisms, including persistent stress responses, neuroendocrine dysregulation, immune imbalance, inflammatory activation, and behavioral changes. Meanwhile, ACE exposure also demonstrates a clear cumulative effect, whereby the risk of future adverse health outcomes increases with the number and types of adversities experienced, and may even rise in a multiplicative manner (3, 4).

In recent years, with the growing recognition of the life-course health perspective, the relationship between childhood adversity and adult health outcomes has become an important focus of interdisciplinary research spanning public health, clinical medicine, psychiatry, epidemiology, and social medicine. A substantial body of evidence has shown that childhood adversity is closely associated with a wide range of adverse health outcomes, particularly mental health problems such as depression, anxiety, and post-traumatic stress disorder. For example, based on quasi-experimental studies, Jessie R Baldwin and colleagues reported a moderate association between childhood maltreatment and mental health problems (Cohen's *d* = 0.56, 95% *CI*: 0.41–0.71) (5). Beyond mental health outcomes, ACEs have also been increasingly recognized as important determinants of a wide range of chronic physical diseases. Evidence suggests that childhood adversity is associated not only with the onset of these conditions, but also with their progression and poorer prognosis across the life course. Among the most consistently reported outcomes are cardiometabolic diseases. For example, a meta-analysis by Jakubowski et al. (6) showed that cumulative childhood adversity was significantly associated with an elevated risk of adult cardiometabolic diseases, with pooled effect sizes of *HR* = 1.42 (95% *CI*: 1.20–1.67) and *OR* = 1.36 (95% *CI*: 1.27–1.46), respectively. Obesity, as a major metabolic disorder, has likewise been frequently linked to ACE exposure. A network analysis by Moriya et al. (7) found that adolescents exposed to physical, sexual, and emotional abuse were more likely to develop overweight or obesity, and this association was particularly pronounced among those who had experienced four or more ACEs. In addition to cardiometabolic outcomes, childhood adversity has been implicated in other chronic conditions affecting multiple physiological systems. A meta-analysis by Bussieres et al. (8) reported that ACE exposure, either alone or in combination with other indirect adverse childhood experiences, significantly increased the risk of chronic pain in adulthood (*aOR* = 1.53, 95% *CI*: 1.42–1.65) and pain-related disability (*aOR* = 1.29, 95% *CI*: 1.01–1.66) (8). Similar associations have also been observed for gastrointestinal disorders such as irritable bowel syndrome (9), autoimmune diseases (10), and certain types of cancer (11). Taken together, these findings suggest that the health consequences of childhood adversity extend far beyond any single disease category. Instead, ACEs appear to confer a broad, cross-system vulnerability that may contribute to chronic disease, multimorbidity, and even premature mortality in adulthood through interconnected pathways involving chronic inflammation, metabolic dysregulation, unhealthy behaviors, and social inequality (11, 12).

From a global public health perspective, childhood adversity and children's exposure to violence have become major issues that can no longer be overlooked. It has been estimated that more than half of all children worldwide—approximately 1 billion children aged 2–17 years—experienced some form of violence in the past year, with prevalence exceeding 50% in Asia, Africa, and North America (13). Reflecting the scale and urgency of this problem, the United Nations Sustainable Development Goals include “ending all forms of violence against children” as Target 16.2. Similarly, World Health Organization member states have committed, through a global plan of action, to strengthening the role of health systems within national multisectoral responses to interpersonal violence, particularly violence against women, girls, and children. Despite this growing policy attention, current prevention and intervention efforts are still often organized separately around issues such as child maltreatment, domestic violence, mental illness, or substance abuse. In reality, however, ACEs rarely occur in isolation. Instead, they frequently co-occur, interact, and accumulate over time, thereby producing compounded long-term health consequences. Supporting this view, Rosalie Broekhof and colleagues found a high degree of ACE co-occurrence already during adolescence, with particularly substantial overlap between neglect and abuse, suggesting that assessing ACEs individually may lead to incomplete or even misleading conclusions regarding their actual distribution and health effects (14). This pattern of “exposure clustering–risk accumulation–intertwined outcomes” indicates that childhood adversity is not merely a child protection issue, but also a broader public health challenge with important implications for chronic disease prevention and control, mental health promotion, and efforts to reduce health inequities (15).

Although research in this field has grown rapidly, the existing literature remains largely fragmented, with most studies focusing on specific disease outcomes, particular types of adversity, or selected subpopulations. As a result, a comprehensive understanding of the overall research landscape—including its knowledge base, major hotspot themes, influential authors and journals, national and institutional collaboration patterns, and evolutionary trends—remains relatively limited. Traditional reviews and meta-analyses are valuable for synthesizing evidence on specific research questions, but they are less suited to revealing the broader disciplinary structure, emerging fronts, and patterns of knowledge development within a field. By contrast, bibliometric methods, combined with quantitative analysis and knowledge-mapping techniques, enable multidimensional assessment of publication trends, core authors, source journals, international collaboration, keyword co-occurrence, and highly cited literature, thereby providing a more comprehensive perspective on the developmental trajectory and research hotspots of this field (16–18). In this context, bibliometric analysis is particularly valuable for research on childhood adversity and chronic diseases. By identifying landmark publications and key evidence sources, it can clarify the intellectual foundations of the field; by analyzing the contributions and collaboration patterns of countries, institutions, and authors, it can reveal the global distribution of research productivity and academic collaboration; and by examining keyword co-occurrence and clustering, it can uncover major hotspots and emerging trends. In doing so, bibliometric analysis can provide useful guidance for future research priorities, interdisciplinary collaboration, and public health policymaking.

## 2 Methods

This study was designed as a bibliometric analysis of published literature metadata and did not involve the evaluation of interventions or the synthesis of clinical outcomes. Therefore, the PICOS framework was not applicable to the objectives and design of this study. Instead, eligibility criteria were defined according to the database source, publication period, language, document type, and relevance to childhood adversity and chronic diseases.

### 2.1 Data sources and search strategy

This study systematically retrieved literature on childhood adversity and chronic diseases from three databases: WoSCC, Scopus, and PubMed (17, 19). The retrieval was restricted to publications written in English. To maximize thematic coverage while minimizing topic contamination, the search strategy was structured into two modules: a childhood adversity module and a chronic disease module. The childhood adversity module included terms such as “adverse childhood experiences,” “childhood adversity,” “early life adversity,” “childhood trauma,” “childhood maltreatment,” and “childhood abuse,” as well as terms explicitly referring to physical, emotional, and sexual abuse occurring during childhood. The chronic disease module focused on major chronic disease categories, including cardiovascular diseases, diabetes, cancer, chronic pain, gastrointestinal diseases, metabolic diseases, and autoimmune diseases. To improve topic specificity, broad expressions that could introduce ambiguity regarding the developmental stage, such as “sexual abuse” alone, were not used directly. Instead, more specific terms, including “child sexual abuse” and “childhood sexual abuse,” were adopted. In this study, “childhood adversity” was used as the broader umbrella term encompassing adverse or traumatic experiences occurring during childhood, whereas “adverse childhood experiences (ACEs)” was retained when referring specifically to the established ACE framework, ACE-based assessments, or studies explicitly using this terminology. Because presenting the complete search strategies for all three databases in the main text would be excessively lengthy, the detailed database-specific search strategies are provided in Supplementary Table 1 (20, 21). For conciseness, the search strategy applied across the three databases was uniformly abbreviated in the study flowchart as “childhood adversity terms AND chronic disease terms.”

The analytical time window was defined as spanning from the earliest relevant publication to December 31, 2025. Records from 2026 were excluded from the formal analysis because database indexing for that year was still ongoing, which could introduce time-truncation bias and affect the stability of annual publication trend analyses.

### 2.2 Literature export and data integration

After retrieval, bibliographic records were exported separately from the three databases. WoSCC records were exported in plain text format with full records, Scopus records in CSV format, and PubMed records in Citation Manager (.nbib) format. All records were then imported into the R 4.5.2 environment and converted into a unified bibliographic data structure using the bibliometrix package to facilitate cross-database integration and analysis (20, 21).

Records from the three databases were first merged into a raw integrated dataset. Automatic cross-database deduplication was not performed at the import stage. Instead, year restriction, document type filtering, and deduplication were conducted sequentially within a unified data-cleaning workflow. This procedure ensured that each step of record exclusion remained traceable and reproducible.

### 2.3 Year restriction, document type filtering, and deduplication

Within the merged raw dataset, records published in 2026 were first removed. Records lacking publication year information were also excluded because their temporal position could not be clearly determined. Document types were then uniformly filtered, retaining only Article and Review records, while excluding conference papers, conference abstracts, editorials, letters, news items, corrections, and other non-research documents to maintain consistency in the knowledge-production attributes of the included literature.

**Figure 1:**
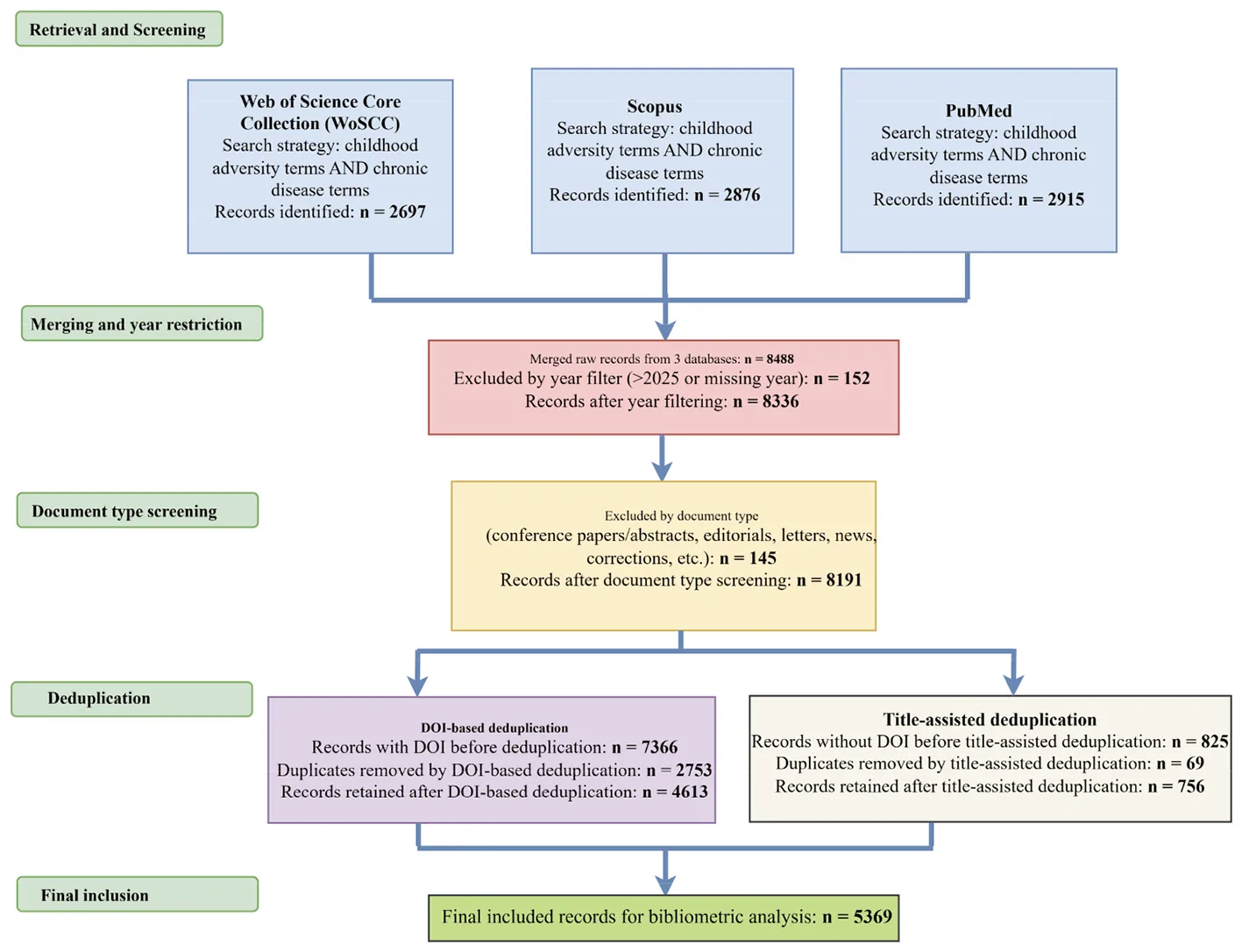
Flowchart of the literature selection process.

Deduplication followed a two-step strategy of “DOI first, title-assisted”. First, DOI fields were standardized by unifying letter case and removing DOI prefixes and redundant spaces. Records containing DOI information were then deduplicated by exact matching based on the standardized DOI. For records without DOI, title-assisted deduplication was subsequently applied. Titles were standardized with respect to letter case, punctuation, and spacing, and duplicate identification was further supported by first author and publication year information to improve the accuracy of deduplication for records lacking DOI. The final cleaned dataset generated through this procedure was used for subsequent bibliometric analyses. The complete screening and deduplication process is shown in Figure 1.

### 2.4 Bibliometric analysis

Based on the cleaned final dataset, bibliometric analyses were conducted to characterize the overall output patterns and knowledge structure of research on childhood adversity and chronic diseases. The main analytical components included annual publication trend analysis, country/region distribution and collaboration analysis, institutional productivity analysis, author productivity analysis, journal distribution analysis, and highly cited literature analysis.

Annual publication trend analysis was used to describe the developmental trajectory of the field over time. Country/region distribution and collaboration analysis was performed to identify the major publishing countries and their international collaboration characteristics. Institutional and author productivity analyses were used to identify the most productive institutions and core author groups, respectively. Journal distribution analysis was conducted to determine the main publication outlets in this field. Highly cited literature analysis was used to identify the representative knowledge base and core studies.

These analyses were primarily conducted using the integrated multi-database dataset, and all associated statistical analyses, data processing, and visualizations were implemented in R. For country-level analysis, *post hoc* standardization was additionally performed for address suffixes, state abbreviations, and synonymous country names in some database exports to reduce potential counting bias caused by differences in address structure.

### 2.5 Keyword co-occurrence and thematic structure analysis

To identify hotspot themes and characterize the thematic structure of research on childhood adversity and chronic diseases, keyword analysis was performed using VOSviewer (22). Keyword sources included author keywords (DE), index keywords (ID), and available subject heading fields (e.g., MH) (23). Before analysis, keyword preprocessing was conducted in R based on the merged and deduplicated final dataset. This process included removal of general demographic and methodological stop words (e.g., “human(s)”, “male”, “female”, “adult”, “review”, and “controlled study”) and harmonization of major synonyms and near-synonyms. For example, adverse childhood experiences, childhood adversity, early life adversity, childhood trauma, and childhood maltreatment were consolidated into a relatively consistent conceptual framework. Disease outcome-related terms, such as cardiovascular disease(s), heart disease, and chronic disease(s), were also moderately standardized according to analytical needs. Cleaned keywords were used for the main text analysis, whereas frequency results of the uncleaned raw keywords were retained in the Supplementary Material to enhance result traceability.

For keyword co-occurrence network construction, the co-occurrence matrix was generated directly in VOSviewer based on document–keyword relationships. To balance information retention with network interpretability, the minimum keyword occurrence threshold was set at 40. This threshold was selected to reduce interference from low-frequency terms, focus the analysis on relatively stable and representative themes, and improve the clarity of cluster identification in the visualization. Under this condition, 297 of 16,075 keywords met the threshold and entered the candidate set. In addition, keyword word clouds and treemaps were generated in R, and the corresponding source data were exported to ensure consistency and reproducibility between the figures and underlying data.

CiteSpace (24, 25), was further used to explore hotspot migration and frontier changes over the most recent decade. To emphasize recent thematic evolution, the time window was restricted to 2015–2025, with 1 year per slice. Term sources included the title, abstract, author keywords (DE), and Keyword Plus information (ID). Network link strength was set to cosine, and node selection was based on the g-index (*k* = 25). On this basis, visual analysis of keyword co-occurrence was conducted (26).

### 2.6 Institutional collaboration network analysis

Institutional collaboration network analysis was performed in VOSviewer. The analysis type was set to Co-authorship, the unit of analysis to Organizations, and the counting method to Full counting. To focus on relatively active contributors and improve the interpretability of the collaboration network, the institutional inclusion threshold was set at a minimum of 20 publications and a minimum of 0 citations. This setting reduced the influence of sporadically appearing institutions with weak connectivity and facilitated clearer identification of the main collaboration structure and cluster patterns among core institutions.

In addition, to limit distortion of the network topology by papers involving extremely large-scale collaborations, the option Ignore documents co-authored by a large number of organizations was selected, and the Maximum number of organizations per document was set to 25. This setting helped reduce artificial inflation of node centrality and link density caused by highly collaborative publications while preserving the main collaboration structure of the field. Under these conditions, 5,867 institutions entered the initial candidate pool, of which 65 met the threshold and were included in the final visualization network.

### 2.7 Citation relationship and citation performance analysis

Citation relationship analysis requires complete, standardized, and structurally comparable citation metadata. Because WoSCC, Scopus, and PubMed differ in citation coverage, reference field structure, and counting rules—and because PubMed does not provide cited-reference metadata with the same degree of structural completeness as WoSCC—citation relationship analysis in this study was conducted primarily based on WoSCC data to reduce bias arising from database heterogeneity and incomplete citation information.

Based on WoSCC data, two citation performance indicators were calculated and presented. *Global Citations* referred to the total number of times a publication had been cited in the entire WoSCC database, reflecting its overall academic influence in the broader literature. *Local Citations* referred to the number of times a publication had been cited by other records within the dataset included in this study, reflecting its knowledge influence and core position within the field of childhood adversity and chronic disease research.

On this basis, the top 10 publications ranked by global citations and the top 10 publications ranked by local citations were identified and reported to recognize studies with high external academic impact as well as central knowledge-hub roles within the field. In addition, to evaluate the distribution of academic influence at a more macroscopic level, total citation frequencies of journals, countries, and authors were also calculated and visualized. The top 10 journals, countries, and authors ranked by total citations were compared to identify the main knowledge dissemination platforms, core national contributors, and representative author groups with high citation impact in this field.

Overall, this study adopted a modular analytical strategy. The integrated multi-database dataset (WoSCC, Scopus, and PubMed) was used to describe academic output patterns, collaboration structures, keyword themes, and the overall knowledge landscape, whereas citation relationship analysis was conducted primarily on WoSCC data. This strategy maximized coverage of the field while taking into account the structural consistency of citation data and the reliability of result interpretation (18).

## 3 Results

### 3.1 Literature screening and inclusion for studies on childhood adversity and chronic diseases

Through systematic retrieval from the WoSCC, Scopus, and PubMed databases, a total of 8,488 records were initially identified. After year-based screening, which excluded records published in 2026 and those lacking publication year information, 8,336 records remained. Document type screening was then conducted, excluding non-research records such as conference papers, editorials, letters, news items, and corrections, resulting in 8,191 records eligible for deduplication. To remove duplicate records, a two-step deduplication strategy was applied. First, DOI-based deduplication was performed among the 7,366 records containing DOI information, through which 2,753 duplicates were removed and 4,613 records were retained. Subsequently, title-assisted deduplication was conducted among the 825 records lacking DOI information, removing an additional 69 duplicates and retaining 756 records. After these two steps, a final total of 5,369 publications were included in the bibliometric analysis. Detailed results are presented in Figure 1.

### 3.2 Annual publication trends in research on childhood adversity and chronic diseases

**Figure 2:**
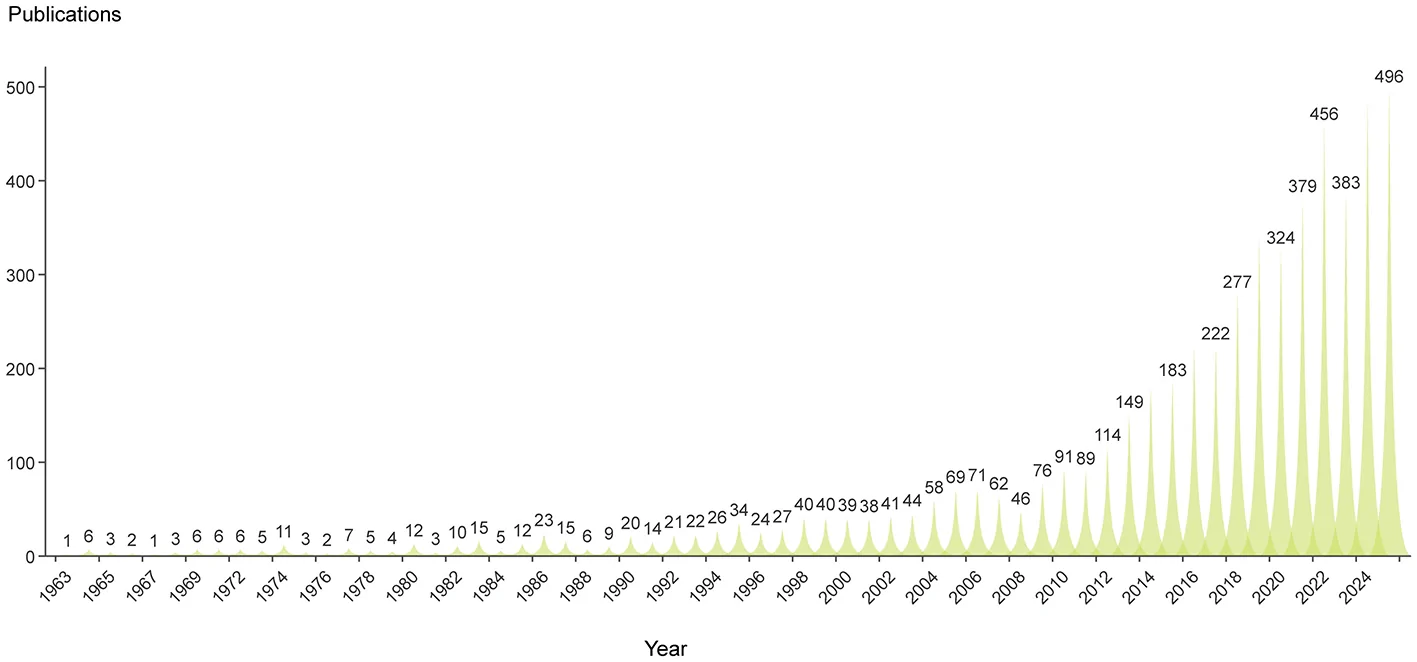
Annual publication trend of research on childhood adversity and chronic diseases from 1963 to 2025.

Figure 2 shows a steady increase in the number of publications in this field over time. From 1963 to the early 1990s, annual publication output remained low, with fewer than 10 papers published per year. Since the mid-1990s, publication output began to increase noticeably, and since 2000, the number of publications has grown substantially. In particular, after 2010, research in this field experienced exponential growth, reaching 496 publications in 2025. This marked increase reflects the growing academic interest in the relationship between childhood adversity and chronic diseases, establishing it as an important research area in recent years.

### 3.3 Country distribution and collaboration patterns

Table 1 presents the top 20 countries ranked by publication output, together with the proportions of multiple-country publications (MCP) and single-country publications (SCP). The United States ranked first with 3,471 publications, far exceeding all other countries. It was followed by the United Kingdom (594 publications) and Canada (382 publications). The United States also demonstrated particularly strong international collaboration, with an MCP proportion of 78.7%, highlighting its dominant position in this field. Among other highly productive countries, Germany (377 publications), Australia (302 publications), and China (222 publications) also ranked prominently. Notably, the Netherlands and France showed high levels of international collaboration, with MCP proportions of 73.3% and 68.5%, respectively.

**Table 1:** Top 20 countries after merging duplicate country labels.

Rank	Country	Publications	SCP	MCP	MCP_percent
1	United States	3,471	739	2,732	78.7
2	United Kingdom	594	200	394	66.3
3	Canada	382	163	219	57.3
4	Germany	377	127	250	66.3
5	Australia	302	91	211	69.9
6	China	222	116	106	47.7
7	Netherlands	187	50	137	73.3
8	France	165	52	113	68.5
9	Italy	139	62	77	55.4
10	Brazil	117	46	71	60.7
11	Sweden	114	28	86	75.4
12	Switzerland	95	17	78	82.1
13	Spain	84	17	67	79.8
14	Denmark	76	18	58	76.3
15	Norway	72	21	51	70.8
16	Finland	69	22	47	68.1
17	Japan	68	37	31	45.6
18	South Africa	66	17	49	74.2
19	Belgium	64	21	43	67.2
20	Israel	57	30	27	47.4

SCP, single-country publications; MCP, multiple-country publications.

Countries such as Switzerland, Spain, and Norway also demonstrated strong international collaborative capacity. Switzerland had the highest MCP proportion at 82.1%, exceeding that of all other countries. South Africa and Belgium likewise showed substantial international collaboration, with MCP proportions of 74.2% and 67.2%, respectively, indicating their important roles in multinational research cooperation. Overall, the table not only reflects publication output across countries in the field of childhood adversity and chronic diseases, but also underscores the critical role of international collaboration. Many of the most productive countries exhibited a strong tendency toward cross-national cooperation, which is essential for advancing global scientific progress and facilitating international knowledge exchange.

### 3.4 Top 10 authors, journals, and institutions in the field of childhood adversity and chronic diseases

**Figure 3:**
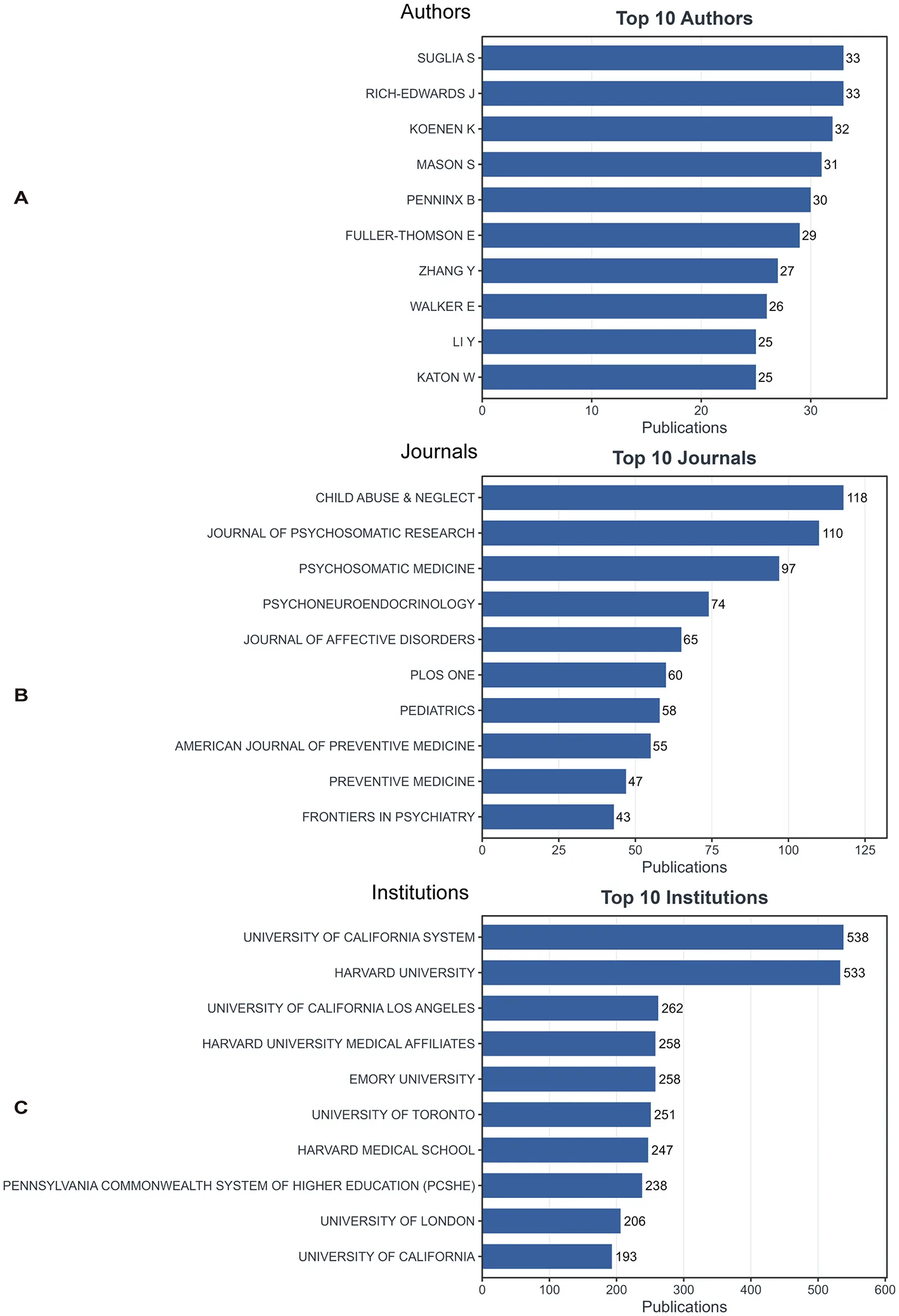
Top 10 authors, journals, and institutions. **(A)** Top 10 authors based on publication count. **(B)** Top 10 journals by the number of publications. **(C)** Top 10 institutions with the highest publication output.

Figure 3 presents the top 10 authors, journals, and institutions contributing to research on childhood adversity and chronic diseases. Figure 3A shows the top 10 authors, with Suglia S. and Rich-Edwards J. ranking first jointly with 33 publications each, followed closely by Koenen K. with 32 publications. Other highly productive authors included Mason S. (31 publications), Penninx B. (30 publications), and Fuller-Thomson E. (29 publications), whose work has provided important theoretical support for understanding the relationship between childhood adversity and chronic diseases.

Figure 3B presents the top 10 journals by publication output. *Child Abuse & Neglect* ranked first with 118 publications, followed by *Journal of Psychosomatic Research* (110 publications) and *Psychosomatic Medicine* (97 publications). These journals have played important roles in disseminating research and advancing scholarship in this field.

Figure 3C lists the top 10 institutions. The University of California System ranked first with 538 publications, followed closely by Harvard University with 533 publications. Other major contributing institutions included the University of California, Los Angeles (262 publications), Harvard University Medical Affiliates (258 publications), and Emory University (258 publications). These institutions have played central roles in promoting research on childhood adversity and chronic diseases.

### 3.5 Keyword co-occurrence and distribution of research hotspots

**Figure 4:**
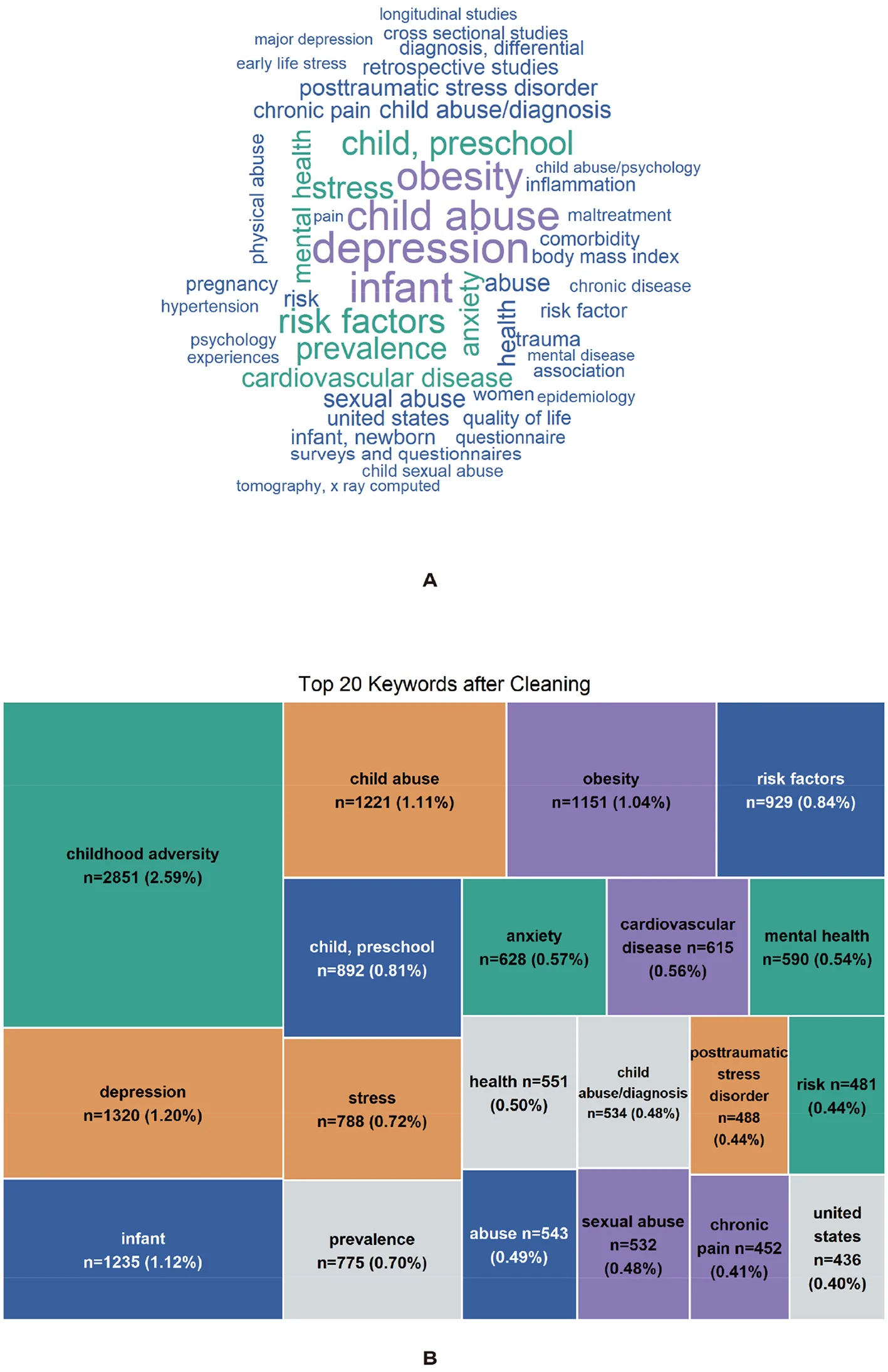
Presents the keyword analysis in the research of childhood adversity and chronic diseases, divided into two parts: **(A)** the keyword word cloud. **(B)** Shows the top 20 keywords after cleaning.

Figure 4 presents the keyword analysis of research on childhood adversity and chronic diseases and consists of two parts: Figure 4A shows the keyword word cloud, and Figure 4B presents the top 20 cleaned keywords.

Figure 4A illustrates the distribution of keywords in this research field. The size of each term is proportional to its frequency of occurrence in the literature. “Child abuse” and “depression” were the largest keywords, reflecting their dominant positions in the field. Other prominent keywords included “obesity,” “mental health,” and “risk factors,” indicating their central importance. In addition, terms such as “sexual abuse,” “cardiovascular disease,” and “prevalence” also appeared in the word cloud, highlighting their relevance in the literature. These keywords were closely associated not only with childhood adversity itself, but also with key health outcomes such as obesity, depression, and cardiovascular disease, suggesting that research in this field has focused on understanding the occurrence of chronic diseases and their relationship with childhood adversity.

Figure 4B lists the 20 most frequent cleaned keywords, together with their frequencies and proportions. “Childhood adversity” ranked first, appearing 2,851 times and accounting for 2.59% of all keyword occurrences, indicating its central role in the literature. This was followed by “child abuse” (1,221 occurrences, 1.11%) and “obesity” (1,151 occurrences, 1.04%). These keywords suggest that research has primarily focused on the long-term effects of childhood adversity on physical and mental health. In addition to these high-frequency terms, keywords such as “risk factors,” “depression,” and “stress” also ranked highly, indicating the broad scope of this field and its coverage of multiple factors related to chronic disease development. Other frequent terms, including “cardiovascular disease,” “mental health,” and “health,” further reflect the major research priorities and interdisciplinary nature of this field. Overall, the two parts of Figure 4 clearly demonstrate the distribution of keywords in research on childhood adversity and chronic diseases, highlighting the importance of topics such as child abuse, mental health, and obesity, as well as the complex relationships between these factors and health outcomes.

### 3.6 Institutional collaboration and keyword co-occurrence networks based on VOSviewer

**Figure 5:**
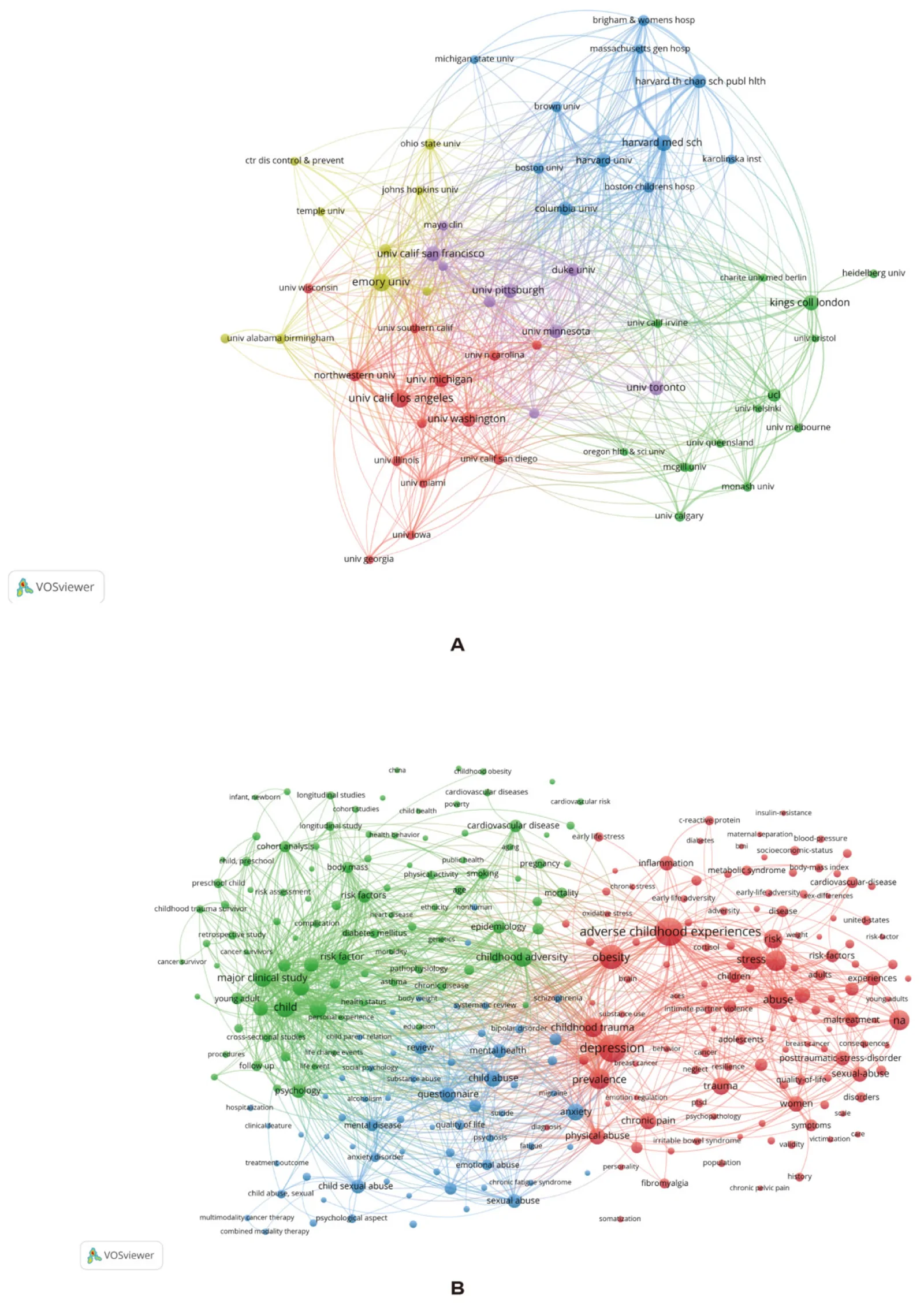
Institution and keyword network analysis in childhood adversity and chronic diseases research. **(A)** Institution collaboration network. **(B)** Keyword co-occurrence network.

Figure 5 presents the network analysis of institutions and keywords in research on childhood adversity and chronic diseases, consisting of two parts: Figure 5A shows the institutional collaboration network, and Figure 5B shows the keyword co-occurrence network.

Figure 5A illustrates the institutional collaboration structure. In this network, each node represents an academic institution, and the size of the node corresponds to its research output in this field, namely the number of publications. Node colors reflect collaboration patterns among institutions, with similarly colored institutions generally showing closer collaboration. The links between nodes indicate collaboration relationships, and the thickness of the links represents collaboration frequency, with thicker lines indicating more frequent collaboration. The University of California System and Harvard University occupied central positions in the network, indicating their leading academic roles in this field. Other major institutions, such as the University of California, Los Angeles and Harvard University Medical Affiliates, were also located near the center of the network, reflecting their strong influence in this area of research.

Figure 5B presents the keyword co-occurrence network generated using VOSviewer. Each node represents a keyword, with node size proportional to its occurrence frequency, while the links between nodes indicate co-occurrence relationships within the same publications. Thicker links represent stronger co-occurrence associations. A total of 297 keywords were grouped into three major thematic clusters, which are summarized in Supplementary Table 2. Cluster 1, comprising 133 keywords, centered on adverse childhood experiences and their multidimensional chronic health consequences, including depression, obesity, inflammation, chronic pain, and posttraumatic stress disorder. Cluster 2 contained 91 keywords and primarily reflected life-course epidemiological research on cardiometabolic risk, health behaviors, cardiovascular disease, mortality, and pregnancy-related outcomes. Cluster 3 consisted of 73 keywords and focused on specific forms of childhood maltreatment and their associations with mental disorders, cognitive impairment, somatic symptoms, and psychiatric–somatic comorbidity. The close connections among “adverse childhood experiences,” “childhood trauma,” “depression,” “obesity,” and “inflammation” suggest that the long-term biopsychosocial consequences of childhood adversity constitute a central research theme in this field.

### 3.7 Keyword clustering based on CiteSpace

**Figure 6:**
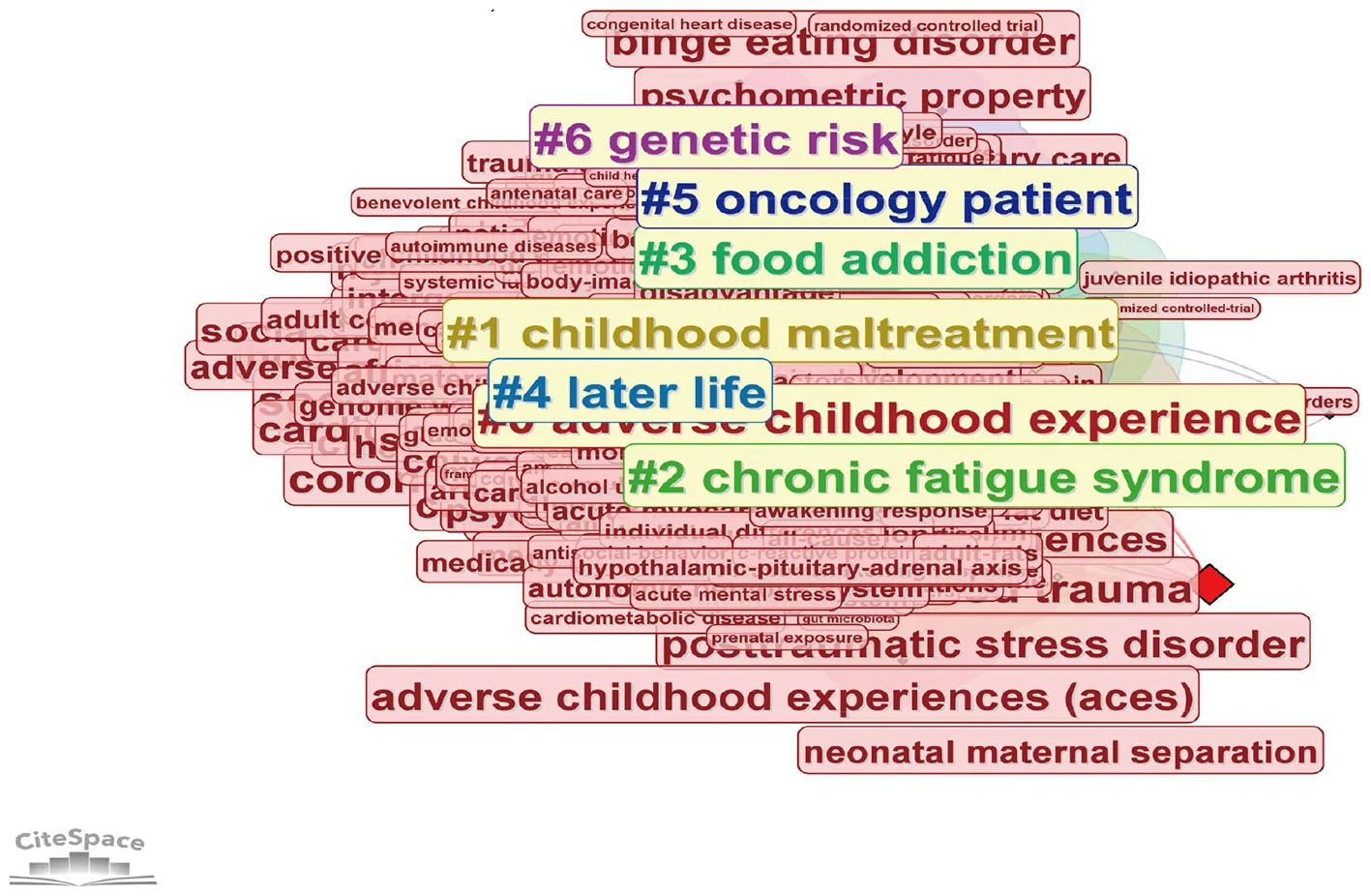
Keyword analysis in childhood adversity and chronic disease research.

Figure 6 presents the keyword clustering network generated using CiteSpace. Node size reflects keyword occurrence frequency, while the spatial proximity and connecting links among nodes indicate thematic and co-occurrence relationships. Seven major keyword clusters were identified, ranging in size from 27 to 106 nodes, with silhouette values between 0.564 and 0.736. The detailed cluster characteristics, representative keywords, and thematic interpretations are presented in Supplementary Table 3. Cluster #0, the largest cluster, broadly represented the chronic health consequences and potential biological pathways associated with childhood adversity, including psychiatric, inflammatory, oncological, and later-life outcomes. Cluster #1 focused on childhood maltreatment, psychological consequences, opioid use, and trauma-informed care. Cluster #2 reflected functional somatic syndromes and the neurobiological effects of early-life stress, particularly chronic fatigue syndrome, irritable bowel syndrome, and visceral hypersensitivity. Cluster #3 centered on food addiction, disordered eating, weight stigma, and obesity-related care, whereas Cluster #4 primarily addressed life-course cardiovascular health and blood-pressure-related outcomes. Cluster #5 highlighted childhood adversity in oncology and breast cancer populations, and Cluster #6 represented emerging research on genetic, reproductive, and anthropometric pathways.

### 3.8 Global citations and local citations

Table 2 lists the top 10 most globally cited publications in the field of childhood adversity and chronic diseases. Ranked by total citation counts, these publications demonstrate broad influence across the global academic community. The 1998 paper by Felitti (27), *Relationship of childhood abuse and household dysfunction to many of the leading causes of death in adults: The Adverse Childhood Experiences (ACE) Study*, ranked first with 13,652 global citations (27). This pioneering study examined the relationship between childhood abuse, household dysfunction, and leading causes of death in adulthood, and has become a cornerstone of ACE research, greatly advancing the development of this field. It was followed by Anda (28) 2006 paper, *The enduring effects of abuse and related adverse experiences in childhood*, which ranked second with 3,079 citations. This study explored the long-term effects of childhood abuse and related adverse experiences on adult health from neurobiological and epidemiological perspectives. Other influential studies included Repetti (29) 2002 paper, *Risky families: Family social environments and the mental and physical health of offspring* (2,162 citations), and Slavich (30) 2014 paper on the social signal transduction theory of depression and inflammation (1,627 citations). Heim C. and McEwen BS were also represented among the top-cited authors; their 2,000 studies on the potential role of cortisol in stress-related bodily disorders and on allostasis made important contributions to understanding the biological mechanisms of stress (31, 32). These influential publications laid the foundation for research on the relationship between childhood adversity and chronic diseases, covering topics such as stress, depression, obesity, and inflammation.

**Table 2:** Top 10 most globally cited publications.

No	First author	Article title	Journal	Year	Global citations
1	Felitti VJ	Relationship of childhood abuse and household dysfunction to many of the leading causes of death in adults. The Adverse Childhood Experiences (ACE) study	Am J Prev Med	1998	13,652
2	Anda RF	The enduring effects of abuse and related adverse experiences in childhood. A convergence of evidence from neurobiology and epidemiology	Eur Arch Psy Clin N	2006	3,079
3	Repetti RL	Risky families: family social environments and the mental and physical health of offspring	Psychol Bull	2002	2,162
4	Slavich GM	From stress to inflammation and major depressive disorder: a social signal transduction theory of depression	Psychol Bull	2014	1,627
5	Heim C	The potential role of hypocortisolism in the pathophysiology of stress-related bodily disorders	Psychoneuroendocrino	2000	1,331
6	Mcewen BS	Allostasis and allostatic load: implications for neuropsychopharmacology	Neuropsychopharmacol	2000	1,269
7	Fazel S	The health of homeless people in high-income countries: descriptive epidemiology, health consequences, and clinical and policy recommendations	Lancet	2014	1,198
8	Hampl SE	Clinical Practice Guideline for the Evaluation and Treatment of Children and Adolescents With Obesity	Pediatrics	2023	1,112
9	Danese A	Childhood maltreatment predicts adult inflammation in a life-course study	P Natl Acad Sci USA	2007	971
10	Danese A	Adverse childhood experiences and adult risk factors for age-related disease: depression, inflammation, and clustering of metabolic risk markers	Arch Pediat Adol Med	2009	952

Table 3 lists the top 10 locally cited publications within the dataset of this study, reflecting their relevance and influence specifically within the field of childhood adversity and chronic diseases. Felitti (27) paper, *Relationship of childhood abuse and household dysfunction to many of the leading causes of death in adults: The Adverse Childhood Experiences (ACE) Study*, was not only widely cited globally, but also ranked first in local citations with 1,003 citations, underscoring its core position in this research field. It was followed by Danese (33) 2014 systematic review and meta-analysis on childhood maltreatment and obesity (239 local citations), and by Dong (34) 2004 study showing how childhood adversity may influence ischemic heart disease through biological mechanisms (228 citations). Other important publications included multiple papers by Anda (28) and Danese (35), which not only provided strong support for understanding childhood adversity, but also laid the groundwork for subsequent research.

**Table 3:** Top 10 most locally cited publications.

No.	First author	Article title	Journal	Year	Local citations
1	Felitti VJ	Relationship of childhood abuse and household dysfunction to many of the leading causes of death in adults. The adverse childhood experiences (ACE) study	Am J Prev Med	1998	1,003
2	Danese A	Childhood maltreatment and obesity: systematic review and meta-analysis	Mol Psychiatr	2014	239
3	Dong MX	Insights into causal pathways for ischemic heart disease: adverse childhood experiences study	Circulation	2004	228
4	Anda RF	The enduring effects of abuse and related adverse experiences in childhood. A convergence of evidence from neurobiology and epidemiology	Eur Arch Psy Clin N	2006	187
5	Danese A	Childhood maltreatment predicts adult inflammation in a life-course study	P Natl Acad Sci USA	2007	173
6	Danese A	Adverse childhood experiences and adult risk factors for age-related disease: depression, inflammation, and clustering of metabolic risk markers	Arch Pediat Adol Med	2009	173
7	Suglia SF	Childhood and adolescent adversity and cardiometabolic outcomes: a scientific statement from the American Heart Association	Circulation	2018	133
8	Davis DA	Are reports of childhood abuse related to the experience of chronic pain in adulthood? A meta-analytic review of the literature	Clin J Pain	2005	113
9	Thomas C	Obesity and type 2 diabetes risk in midadult life: the role of childhood adversity	Pediatrics	2008	112
10	Hemmingsson E	Effects of childhood abuse on adult obesity: a systematic review and meta-analysis	Obes Rev	2014	110

These findings highlight the studies with the greatest local impact in this field, particularly those addressing the long-term health consequences of childhood adversity, and indicate that major research hotspots have concentrated on the links between violence, obesity, heart disease, and other chronic conditions in relation to childhood traumatic experiences.

### 3.9 Top 10 cited journals, authors, and countries/regions in the field of childhood adversity and chronic diseases

**Figure 7:**
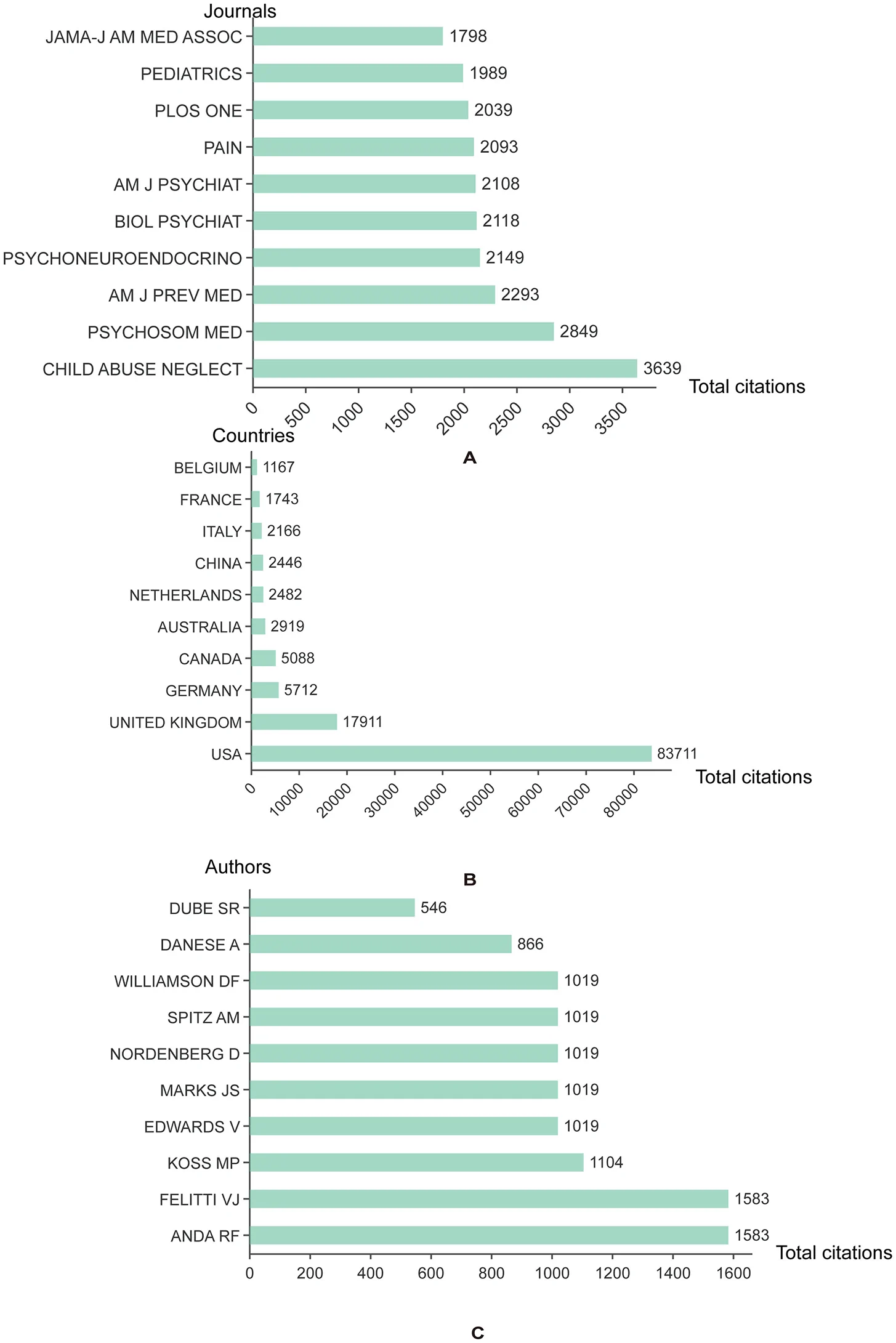
Top 10 journals **(A)**, countries **(B)**, and authors **(C)** by total citations in the field of childhood adversity and chronic diseases.

Figure 7 presents the citation rankings of the top 10 journals, authors, and countries/regions in the field of childhood adversity and chronic diseases, divided into three parts: Figure 7A shows the top 10 journals by citation count, Figure 7B the top 10 authors by citation count, and Figure 7C the top 10 countries/regions by citation count.

In Figure 7A, *Child Abuse & Neglect* ranked first with 3,639 citations, substantially exceeding the other journals, indicating its major influence in this field. It was followed by *Psychosomatic Medicine* (2,849 citations) and *American Journal of Preventive Medicine* (2,293 citations), both of which also showed strong influence. Other top-ranked journals included *Biological Psychiatry, Journal of Pain*, and *PLOS ONE*, which have all served as important academic platforms for researchers in this area.

In Figure 7B, the United States was by far the most cited country, with 83,711 citations, greatly surpassing all others and demonstrating its leading role in this field. It was followed by the United Kingdom (17,911 citations) and Germany (5,712 citations), both of which were also major contributors. Other highly ranked countries included Canada (5,088 citations), Australia (2,919 citations), and the Netherlands (2,482 citations), all of which have played important roles in international collaboration and multinational research.

In Figure7C, Felitti VJ and Anda RF ranked jointly first, each with 1,583 citations, underscoring their central positions in this field. They were followed by Koss MP (1,104 citations), and then Edwards V, Marks JS, Nordenberg D, Spitz AM, and Williamson DF, each with 1,019 citations. These authors have made major contributions to research on childhood adversity and chronic diseases and have substantially advanced the academic development of the field.

Overall, Figure 7 not only identifies the most influential journals, authors, and countries in research on childhood adversity and chronic diseases, but also highlights the leading role of the United States and the critical contributions of these journals and authors to advancing global research.

## 4 Discussion

### 4.1 Overview of the main findings

Based on data from WoSCC, Scopus, and PubMed, this study constructed a knowledge map of research on childhood adversity and chronic diseases up to 2025, and systematically analyzed the field in terms of publication trends, national and institutional collaboration, knowledge base, and thematic evolution. Overall, the number of publications in this field has continued to increase, indicating that the association between childhood adversity and chronic diseases has become a sustained topic of scholarly interest and has gradually developed into an interdisciplinary research area with stable attention. The relatively large number of studies ultimately included after screening and deduplication also indirectly reflects the substantial accumulation of research in this field.

In terms of national contributions, the United States performed prominently in both publication output and citation impact, while also demonstrating a high level of international collaboration, indicating its central role in knowledge production and collaboration networks in this field. The United Kingdom, Germany, Canada, Australia, and China followed, suggesting that this research area has developed clear characteristics of multinational participation and multicenter advancement. At the institutional level, the University of California System and Harvard University occupied important positions in the collaboration network, highlighting the key role of leading universities and medical research institutions in driving the development of this field.

From the perspective of research themes, keyword co-occurrence and burst analyses showed that research on childhood adversity and chronic diseases has mainly focused on themes such as *childhood adversity, child abuse, depression, obesity, stress, risk factors*, and *cardiovascular disease*, indicating that current research priorities are centered on exposure types of childhood adversity, mental health outcomes, metabolic abnormalities, stress mechanisms, and cardiovascular risk. At the same time, related themes have continued to expand toward more specific directions such as chronic pain, chronic fatigue, and intergenerational effects, suggesting that the content of this field is becoming increasingly refined and in-depth.

### 4.2 Knowledge base and research hotspots: from ACE association validation to mechanistic pathways and a multimorbidity framework

The structure of highly cited publications suggests that the knowledge base of this field has followed a relatively clear evolutionary trajectory of “foundation–expansion–integration.” Early research was anchored by the ACE framework proposed and empirically established by Felitti et al. Based on a large sample of adults undergoing health appraisal within a health maintenance organization (HMO), this landmark study was the first to integrate multiple adverse experiences—such as childhood abuse and household dysfunction—into a unified analytical framework, and used logistic regression analyses to systematically demonstrate a significant dose–response relationship between cumulative ACE exposure and adult risk behaviors, health status, and multiple disease outcomes. As the number of ACE categories increased, the risks of alcoholism, drug abuse, depression, suicide attempts, smoking, severe obesity, ischemic heart disease, cancer, chronic lung disease, and liver disease all rose in a graded manner, thereby establishing the core analytical logic of “cumulative exposure–increasing risk” (15, 27). Building on this, Dong et al. further showed that different types of ACEs are not independent of one another, but instead exhibit substantial co-occurrence and clustering. Their study found that approximately two-thirds of participants had experienced at least one type of ACE, and that once one ACE was present, the probability of exposure to additional ACEs increased significantly, with adjusted odds ratios ranging from 2.0 to 17.7. Moreover, the observed number of participants with high ACE scores was significantly greater than expected under the assumption of independence (*p* < 0.0001), thereby statistically confirming the clustered distribution of ACE exposure (36). Together, these two classic studies laid the foundation for the field: the former answered whether ACEs are related to adult health risks, while the latter clarified how ACEs cluster in real life. This shift helped move the research paradigm from “single adversity–single outcome” toward a broader perspective of “exposure clustering–risk accumulation–multiple interrelated outcomes,” providing a core theoretical basis for subsequent studies on mechanistic pathways, expansion of disease spectra, and multimorbidity. On this basis, Anda et al. further extended the ACE framework from the perspectives of public health and global surveillance, emphasizing that ACEs should not be understood merely as several discrete adverse childhood events, but rather as cumulative stress exposures with broad, long-term, and multisystem health consequences. The burden of disease associated with ACEs, they argued, arises more from the chronic cumulative effect of multiple adversities than from any single exposure alone. This study also highlighted that the explanatory strength of the ACE score lies precisely in its ability to capture the co-occurrence of multiple adversities and their cumulative effects on neurodevelopment, stress-response systems, inflammation, behavioral patterns, and ultimately adult disease, disability, and premature mortality, thereby advancing ACE research from association testing toward mechanistic explanation, primary prevention, and a global public health governance framework (2). Subsequent systematic reviews and meta-analyses further demonstrated stable dose–response relationships between ACEs and smoking, problematic alcohol use, mental disorders, suicidal behaviors, and a wide range of chronic diseases, with risks rising particularly sharply among those exposed to four or more ACEs. These findings indicate that classic ACE research established not only a classification system of exposures, but also an explanatory framework grounded in a life-course perspective and emphasizing cumulative damage effects (4, 12, 37, 38).

If early studies mainly addressed whether childhood adversity is associated with adult disease, later highly cited studies increasingly focused on how such associations occur. At the mechanistic level, the framework of allostasis and allostatic load proposed by Danese and McEwen is particularly representative. According to this framework, the body does not maintain stability through static equilibrium, but rather through dynamic adjustments of the nervous, endocrine, and immune systems to achieve “stability through change” (allostasis). However, when childhood adversity is chronic, repeated, and insufficiently buffered by protective support, these originally adaptive stress responses may become sustained physiological “wear and tear,” namely allostatic load, and may even progress to allostatic overload (37). Specifically, adversity exposure during critical developmental windows may induce so-called “biological embedding.” On the one hand, it may alter threat detection, emotion regulation, and executive control through changes in the amygdala–hippocampus–prefrontal cortex network, shaping a neural processing style characterized by heightened vigilance and diminished sense of safety (37). On the other hand, chronic activation of the hypothalamic–pituitary–adrenal (HPA) axis and sympathetic nervous system may lead to abnormal cortisol secretion patterns, dysregulated stress responses, elevated inflammatory activity, and altered immune regulation; studies have shown that individuals with childhood trauma exhibit significantly elevated baseline peripheral levels of CRP, IL-6, and TNF-α (39). More importantly, these systems do not operate independently, but form mutually reinforcing feed-forward loops under prolonged stress; for example, inflammation may further exacerbate HPA-axis dysregulation, while chronic neuroendocrine activation may worsen metabolic and immune imbalance. As a result, the impact of childhood adversity extends beyond short-term psychological responses and instead reshapes the developmental trajectories of neural, endocrine, and immune systems over the long term, producing a cross-system state of heightened vulnerability in adulthood and ultimately increasing the risks of cardiovascular disease, metabolic disorders, depression, post-traumatic stress disorder, and even accelerated biological aging (38). The contribution of Danese and McEwen lies not only in proposing a framework for how ACEs “get under the skin,” but also in showing that the relationship between childhood adversity and adult chronic disease is not a simple linear association, but a life-course process jointly driven by early adaptation, long-term dysregulation, and systemic wear and tear.

Relatedly, the theory of “biological embedding” proposed by Berens et al. further emphasizes that adversity can leave enduring marks on neurodevelopment, endocrine regulation, immune inflammation, and behavioral adaptation, thereby enabling childhood exposures to shape adult health across time (1). At the same time, the toxic stress model suggests that when intense and frequent adversity persists without the buffering effect of supportive relationships, children's stress-response systems may be durably reprogrammed, laying a biological foundation for later multisystem disease risk (38). A recent meta-analytic structural equation model further indicates that inflammation is likely to mediate the association between ACEs and adult depression, suggesting that mental symptoms and physical disease risks are not separate phenomena, but may share upstream stress-related biological mechanisms.

Recent disease-specific systematic reviews published between 2020 and 2024 have further linked general frameworks of biological embedding, allostatic load, and chronic inflammation to specific chronic health outcomes. In the cardiovascular domain, a systematic review of 12 studies suggested a possible association between adverse childhood experiences and elevated blood pressure or hypertension among adult women, although the findings were inconsistent. Significant associations were reported in approximately 60% of studies using self-reported hypertension but in only 30% of those using objectively measured blood pressure, while several studies identified inverse associations (40). This heterogeneity may be attributable to differences in blood-pressure measurement, ACE assessment, and population characteristics. These findings suggest that persistent activation of stress-response systems, autonomic dysregulation, chronic inflammation, and the accumulation of adverse health behaviors should be considered when interpreting the long-term cardiovascular vulnerability associated with childhood adversity. In the metabolic domain, a systematic review and meta-analysis by Zhu et al. (41), including 49 studies, found that exposure to any ACE was associated with a 22% higher risk of diabetes in adulthood, whereas exposure to four or more ACEs was associated with a 44% higher risk. Specific exposures, including physical abuse, sexual abuse, verbal abuse, and childhood economic hardship, were also associated with diabetes risk. A subsequent global meta-analysis of 71 studies further demonstrated significant associations between ACE exposure and both obesity and overweight. The pooled odds ratio was 1.42 for obesity and 1.16 for overweight, while exposure to four or more ACEs was associated with more than a twofold increase in the odds of obesity (42). These findings are consistent with potential pathways involving prolonged neuroendocrine dysregulation, impaired glucose and lipid metabolism, sleep disturbance, chronic inflammation, and maladaptive eating behaviors. In relation to chronic pain, the systematic review and meta-analysis by Bussieres et al. included 85 studies, of which 57 were incorporated into the quantitative synthesis. Direct ACE exposure was associated with a higher likelihood of chronic pain in adulthood, with a pooled adjusted odds ratio of 1.45. Childhood physical abuse was also associated with both chronic pain and pain-related disability, and a clear cumulative pattern was observed, with exposure to four or more ACEs associated with nearly twice the odds of chronic pain (8). A subsequent systematic review of 13 studies further suggested that cumulative childhood abuse and neglect may contribute to persistent pain through emotional dysregulation, sustained hyperarousal, and altered pain processing related to post-traumatic stress disorder or complex post-traumatic stress disorder (43). Collectively, these recent reviews indicate that childhood adversity may influence multiple chronic diseases through interconnected neuroendocrine, autonomic, immune-inflammatory, metabolic, behavioral, and pain-processing pathways. However, the relative contribution of these mechanisms is likely to vary across conditions. Because most available evidence is observational, these findings support biologically plausible pathophysiological pathways rather than establishing definitive causal mechanisms.

At the level of disease outcomes, research in this field has gradually expanded from association testing focused mainly on mental health problems to a broader spectrum of chronic diseases and their overlapping outcomes. On the one hand, the relationship between childhood adversity and later psychopathology remains a central line of inquiry. A systematic review of quasi-experimental studies by Baldwin et al. showed that even after controlling for familial confounding, the association between childhood maltreatment and adult mental health problems remained, suggesting that this relationship cannot be fully explained by shared family background (5). On the other hand, an increasing number of systematic reviews and meta-analyses have demonstrated stable associations between ACE exposure and a variety of chronic physical diseases, including obesity, type 2 diabetes, cardiometabolic diseases, and chronic pain. For example, Jakubowski et al. (6) found that cumulative childhood adversity was significantly associated with adult cardiometabolic disease risk; Danese et al. (33) reported that childhood maltreatment was associated with increased obesity risk; and studies by Huang et al. (44) and Zhu et al. (41) further supported a stable link between ACEs and adult diabetes risk. Bussieres et al. (8) likewise showed that single or cumulative ACE exposure significantly increases the risk of chronic pain and pain-related disability in adulthood. These findings are highly consistent with the high-frequency keywords identified in this study, such as *depression, obesity, cardiovascular disease, mental health*, and *risk factors*, indicating that the field has evolved from single-disease association studies to an integrated disease-spectrum approach encompassing mental, metabolic, cardiovascular, and pain-related systems.

Even more notably, recent research has begun to shift from “single disease risk” toward identifying “multimorbidity” and “integrated risk networks.” The latest systematic reviews and cohort studies suggest that ACEs not only increase the risk of specific diseases, but are also closely associated with the earlier onset, faster accumulation, and greater burden of multimorbidity in adulthood (45, 46). This means that the long-term effects of ACEs do not merely predispose individuals to one particular disease, but are more likely to shape a cross-system vulnerable state through multiple pathways, including persistent inflammatory activation, metabolic dysregulation, sleep and emotional disturbances, the accumulation of unhealthy behaviors, and the continuation of social disadvantage. Combined with the observation in this study that the top 10 locally cited publications concentrated more heavily on evidence synthesis related to obesity/cardiometabolic outcomes and trauma exposure, it can be inferred that the core concern within the field has shifted from “whether an association exists” to “through which mechanisms it occurs, in whom or under what conditions it is stronger, which outcomes are most robust, and which intervention points are most critical.” In this sense, childhood adversity research is gradually moving from early association-testing studies toward mechanism-stratified, risk-stratified, and intervention-oriented research, thereby providing a stronger evidence base for trauma-informed care, early-life screening, and multisectoral collaborative intervention.

### 4.3 Clinical and translational implications: from evidence standards to stratified prevention and implementation feasibility

The pathway of knowledge evolution revealed by this study—from early association testing to mechanistic pathways and evidence integration—also suggests that if this field is to truly enter the stage of clinical and public health translation, the key task is no longer merely to accumulate more evidence that “ACEs are associated with chronic disease,” but rather to clarify which patterns of exposure confer the highest risk, which outcomes are most amenable to intervention, and which strategies can be implemented sustainably in real-world settings.

First, the heterogeneity of exposure–outcome relationships requires a shift from a “one-size-fits-all” approach toward risk stratification and precision prevention. Childhood adversity is not a single exposure; it varies substantially in terms of frequency, severity, developmental timing, subjective perception, and perpetrator role. Correspondingly, adult chronic disease outcomes are distributed across multiple systems and often coexist as multimorbidity. Methodological reviews have pointed out that current ACE research exhibits substantial heterogeneity in the definition and operationalization of exposure dimensions. Summarizing all exposures solely through a total ACE score risks obscuring the true differences in risk associated with distinct adversity patterns. Systematic reviews on ACEs and multimorbidity also suggest a stable association between cumulative adversity and adult multiple chronic disease burden, supporting a future analytical framework that moves beyond “cumulative counts” toward “exposure pattern identification–risk stratification–precision intervention” (12, 45, 47).

Second, the standardization of measurement and reporting determines the upper limit of evidence quality. If studies differ substantially in ACE measurement tools, exposure definitions, chronic disease outcome classifications, and mediator selection, not only will comparability in meta-analyses be weakened, but the stability of keyword clustering and thematic evolution analyses will also be reduced. Existing reviews have clearly shown substantial variation in ACE measurement in terms of study populations, item composition, dimensional definitions, and scoring methods. Studies on core outcome sets in multimorbidity research further suggest that quality of life, mental health, and mortality should be regarded as basic core outcomes; in low- and middle-income country (LMIC) settings, outcomes with greater translational relevance—such as health-risk behaviors, treatment adherence, and out-of-pocket costs—should also be incorporated (47–49). Accordingly, future research on ACEs and chronic diseases should advance two forms of standardization in parallel: first, the development of a core indicator system for childhood adversity that can be mapped across cohorts; and second, the establishment of core outcome sets applicable to chronic disease and multimorbidity research, thereby improving the usability of evidence for synthesis and policy translation.

Third, evidence demand is shifting from “whether associations exist” to “which strategies are more effective,” meaning that comparative effectiveness research and implementation evidence need to be advanced simultaneously. Existing systematic reviews of ACE-related interventions suggest that some multicomponent interventions within pediatric healthcare systems may improve child outcomes, but evidence remains limited as to whether routine ACE screening itself can produce long-term benefits in mental health, service linkage, and cost-effectiveness. In particular, systematic reviews of ACE screening in children indicate that current evidence only modestly supports improved adversity identification and referral, while direct evidence for improvements in child or caregiver mental health outcomes remains lacking. Reviews targeting the general population similarly conclude that evidence is currently insufficient to support widespread routine screening. At the same time, implementation studies of trauma-informed care suggest that what truly determines translational effectiveness is not simply whether screening is conducted, but also system-level implementation conditions such as leadership support, staff training, cross-sector collaboration, screening and referral workflows, quality assessment, and funding allocation (50–53). Therefore, future research should strengthen longitudinal causal inference, comparative effectiveness designs, and implementation science, so as to build a closed-loop evidence chain from screening and stratification to intervention and follow-up.

Fourth, international collaboration and equity represent the “last mile” of translation into practice. Although this field has already formed a sizable international collaboration network, caution is needed to avoid further widening health inequalities during translation into clinical and public health practice because of differences in tools, resources, and implementation capacity. Research in health equity implementation science has emphasized that unless resource accessibility, contextual differences, and the inclusion of disadvantaged populations are incorporated into study design, translational benefits may preferentially accrue to high-resource settings. Reviews focusing on LMIC contexts further show that currently available tools for identifying ACE-related risk factors still have limited adaptability in primary care and resource-constrained settings (54). This implies that future research on ACEs and chronic diseases requires not only more data from low- and middle-income countries and resource-limited regions, but also more context-sensitive implementation studies to improve the external validity, equity, and generalizability of the evidence.

### 4.4 Limitations and future directions: the boundaries of bibliometrics and a roadmap for evidence upgrading

Although the integration of multiple databases enhanced the breadth of coverage, the following limitations should still be interpreted with caution. First, multi-database heterogeneity may introduce residual noise. Although bibliometrics is an effective tool for analyzing large-scale scientific literature, methodological studies have shown that its results depend heavily on database selection, data cleaning, field harmonization, and transparency of the analytical workflow. In multi-source database studies, the standardization of author names, institution names, keywords, and early online publications is especially critical; otherwise, synonymous concepts may be split, institutions or countries may be misclassified, and network structures may become unstable (55, 56).

Second, citation indicators are subject to time lag and classical citation bias. Highly cited publications typically enjoy a longer window for dissemination and accumulation, and therefore are more likely to reflect the “knowledge base” of a field rather than its “latest frontier.” Studies of highly cited publications have likewise pointed out that classic papers are usually earlier publications that have already achieved broad diffusion. Accordingly, the global and local highly cited results in this study are more appropriately understood as foundational knowledge nodes in the field, rather than being directly equated with the most active current research frontiers.

Third, topic contamination and semantic drift cannot be entirely avoided, and bibliometrics cannot replace causal inference or effect-size evaluation. High-frequency terms such as *stress, inflammation*, and *mental health* are themselves shared across disciplines and may therefore pull marginal topics into the network structure. At the same time, bibliometrics fundamentally depicts relationships of knowledge production, dissemination, and linkage, rather than the causal strength between exposures and outcomes or the net clinical benefit of interventions. Methodological research has therefore emphasized that bibliometric findings are more suitable for identifying field structure, knowledge bases, and thematic shifts, but cannot substitute for systematic reviews, meta-analyses, or causal inference studies when addressing questions such as “how large is the effect,” “is the relationship causal,” or “which intervention is most effective.”

Given these limitations, future research may upgrade the evidence base along four major directions. First, longitudinal cohorts, family-based designs, natural experiments, and quasi-experimental studies should be strengthened to improve causal identification. Second, the field should promote standardization and shared infrastructure for ACE exposure measurement and for reporting chronic disease and multimorbidity outcomes. Third, comparative effectiveness research, implementation science, and health equity frameworks should be more systematically incorporated into the research agenda, with the aim of developing a closed-loop evidence chain that is screenable, stratifiable, intervenable, and evaluable for high-risk subgroups. Fourth, artificial intelligence and machine-learning methods are beginning to influence bibliometric and evidence-synthesis workflows in public health research. Active-learning systems can assist researchers in prioritizing potentially relevant records during literature screening, thereby reducing the manual workload associated with large datasets (57). Meanwhile, natural language processing, document embeddings, and transformer-based topic-modeling approaches can support semantic clustering, theme extraction, and the identification of emerging or interdisciplinary research topics that may not be fully captured by conventional keyword co-occurrence analysis (58). However, these approaches should be regarded as decision-support tools rather than substitutes for expert judgment. Future applications should retain human verification and clearly report model selection, training data, parameter settings, performance assessment, and procedures for managing algorithmic bias and reproducibility.

Overall, research on childhood adversity and chronic diseases is moving from a phase of “evidence accumulation” toward one of “mechanistic deepening and translational orientation.” The key task for the future is not to repeatedly confirm associations, but rather to translate the field's knowledge advantages into operational and equitably disseminated public health benefits through stratification, standardization, methodological innovation, and implementation optimization.

## 5 Conclusion

Based on the Web of Science Core Collection (WoSCC), Scopus, and PubMed, this study systematically integrated and cleaned the literature on childhood adversity and chronic diseases and constructed a knowledge map of the field up to 2025. Overall, the results indicate that publication output in this field has shown a sustained long-term upward trend, and that the research focus has gradually expanded from early association testing to the elucidation of mechanistic pathways and evidence integration, suggesting that childhood adversity has become a core and consistently recognized topic in life-course health research. From the perspective of global contributions, the United States occupies a clearly dominant position in both publication output and citation impact, and shows a high proportion of MCP, reflecting its hub role in the international collaboration network. The United Kingdom, Germany, Canada, Australia, and China form a second tier, indicating that the field has developed into an internationally collaborative and multicenter research landscape. At the institutional level, the University of California System and Harvard University occupy central positions in the collaboration network, underscoring the critical role of leading research institutions in knowledge production and dissemination. Keyword analysis showed that themes such as *childhood adversity/child abuse, depression, obesity, stress, risk factors*, and *cardiovascular disease* constitute the major research hotspots, demonstrating clear intersections between mental health and cardiometabolic outcomes, while also revealing an extension toward newer directions such as chronic pain, chronic fatigue syndrome, and intergenerational transmission. The structure of highly cited publications further confirms that the ACE framework and the classic studies linking ACEs with major chronic disease risks in adulthood form the core knowledge base of this field and continue to shape subsequent research agendas. In summary, this study reveals, from a macro-level perspective, the knowledge base, hotspot themes, and collaboration patterns of research on childhood adversity and chronic diseases. Future research should further promote the standardization of exposure and outcome measurement, strengthen longitudinal and causal inference evidence, develop risk-stratified precision prevention frameworks, and enhance implementation science and cross-regional collaboration, so as to improve the operability and equity of research findings in clinical and public health decision-making.

## Data Availability

The original contributions presented in the study are included in the article/Supplementary material, further inquiries can be directed to the corresponding author.
