# Supplementary material for: Global landscape, research hotspots, and evolutionary trends in childhood adversity and chronic disease research: a bibliometric analysis based on three databases
Source: Front Public Health. 2026 Aug 25;14:1862548. doi: 10.3389/fpubh.2026.1862548 (PMC13550130; doi:10.3389/fpubh.2026.1862548)
Supplement: Supplementary file 1 [file Table_1.docx]

**STable 1. Search strategies used in the three databases**

| **Database** | **Search strategy** |
| --- | --- |
| **Web of Science** | TS=(  "adverse childhood experiences" OR  "childhood adversity" OR  "early life adversity" OR  "childhood trauma" OR  "childhood maltreatment" OR  "childhood abuse" OR  "child sexual abuse" OR  "childhood sexual abuse" OR  "child physical abuse" OR  "childhood physical abuse" OR  "child emotional abuse" OR  "childhood emotional abuse" ) AND TS=(  "cardiovascular disease" OR  "cardiovascular diseases" OR  "heart disease" OR  "hypertension" OR  "coronary artery disease" OR  "stroke" OR  "myocardial infarction" OR  "heart failure" OR  "diabetes" OR  "type 1 diabetes" OR  "type 2 diabetes" OR  "cancer" OR  "breast cancer" OR  "lung cancer" OR  "colorectal cancer" OR  "leukemia" OR  "chronic pain" OR  "fibromyalgia" OR  "chronic fatigue syndrome" OR  "irritable bowel syndrome" OR  "inflammatory bowel disease" OR  "ulcerative colitis" OR  "Crohn's disease" OR  "gastroesophageal reflux disease" OR  "metabolic syndrome" OR  "obesity" OR  "autoimmune disease" OR  "autoimmune diseases" OR  "rheumatoid arthritis" OR  "lupus" OR  "multiple sclerosis" ) |
| **Scopus** | TITLE-ABS-KEY(  "adverse childhood experiences" OR  "childhood adversity" OR  "early life adversity" OR  "childhood trauma" OR  "childhood maltreatment" OR  "childhood abuse" OR  "child sexual abuse" OR  "childhood sexual abuse" OR  "child physical abuse" OR  "childhood physical abuse" OR  "child emotional abuse" OR  "childhood emotional abuse" ) AND TITLE-ABS-KEY(  "cardiovascular disease" OR  "cardiovascular diseases" OR  "heart disease" OR  "hypertension" OR  "coronary artery disease" OR  "stroke" OR  "myocardial infarction" OR  "heart failure" OR  "diabetes" OR  "type 1 diabetes" OR  "type 2 diabetes" OR  "cancer" OR  "breast cancer" OR  "lung cancer" OR  "colorectal cancer" OR  "leukemia" OR  "chronic pain" OR  "fibromyalgia" OR  "chronic fatigue syndrome" OR  "irritable bowel syndrome" OR  "inflammatory bowel disease" OR  "ulcerative colitis" OR  "Crohn's disease" OR  "gastroesophageal reflux disease" OR  "metabolic syndrome" OR  "obesity" OR  "autoimmune disease" OR  "autoimmune diseases" OR  "rheumatoid arthritis" OR  "lupus" OR  "multiple sclerosis" ) |
| **PubMed** | (  "Adverse Childhood Experiences"[Title/Abstract] OR  "adverse childhood experiences"[MeSH Terms] OR  "childhood adversity"[Title/Abstract] OR  "early life adversity"[Title/Abstract] OR  "childhood trauma"[Title/Abstract] OR  "childhood maltreatment"[Title/Abstract] OR  "child abuse"[MeSH Terms] OR  "childhood abuse"[Title/Abstract] OR  "child sexual abuse"[Title/Abstract] OR  "childhood sexual abuse"[Title/Abstract] OR  "child physical abuse"[Title/Abstract] OR  "childhood physical abuse"[Title/Abstract] OR  "child emotional abuse"[Title/Abstract] OR  "childhood emotional abuse"[Title/Abstract] ) AND (  "Cardiovascular Diseases"[MeSH Terms] OR  "cardiovascular disease"[Title/Abstract] OR  "cardiovascular diseases"[Title/Abstract] OR  "heart disease"[Title/Abstract] OR  "Hypertension"[MeSH Terms] OR  "hypertension"[Title/Abstract] OR  "coronary artery disease"[Title/Abstract] OR  "Stroke"[MeSH Terms] OR  "stroke"[Title/Abstract] OR  "myocardial infarction"[Title/Abstract] OR  "Heart Failure"[MeSH Terms] OR  "heart failure"[Title/Abstract] OR  "Diabetes Mellitus"[MeSH Terms] OR  "diabetes"[Title/Abstract] OR  "type 1 diabetes"[Title/Abstract] OR  "type 2 diabetes"[Title/Abstract] OR  "Neoplasms"[MeSH Terms] OR  "cancer"[Title/Abstract] OR  "breast cancer"[Title/Abstract] OR  "lung cancer"[Title/Abstract] OR  "colorectal cancer"[Title/Abstract] OR  "leukemia"[Title/Abstract] OR  "Chronic Pain"[MeSH Terms] OR  "chronic pain"[Title/Abstract] OR  "fibromyalgia"[Title/Abstract] OR  "chronic fatigue syndrome"[Title/Abstract] OR  "Irritable Bowel Syndrome"[MeSH Terms] OR  "irritable bowel syndrome"[Title/Abstract] OR  "Inflammatory Bowel Diseases"[MeSH Terms] OR  "inflammatory bowel disease"[Title/Abstract] OR  "ulcerative colitis"[Title/Abstract] OR  "Crohn's disease"[Title/Abstract] OR  "Gastroesophageal Reflux"[MeSH Terms] OR  "gastroesophageal reflux disease"[Title/Abstract] OR  "Metabolic Syndrome"[MeSH Terms] OR  "metabolic syndrome"[Title/Abstract] OR  "Obesity"[MeSH Terms] OR  "obesity"[Title/Abstract] OR  "Autoimmune Diseases"[MeSH Terms] OR  "autoimmune disease"[Title/Abstract] OR  "autoimmune diseases"[Title/Abstract] OR  "Rheumatoid Arthritis"[MeSH Terms] OR  "rheumatoid arthritis"[Title/Abstract] OR  "Lupus Erythematosus, Systemic"[MeSH Terms] OR  "lupus"[Title/Abstract] OR  "Multiple Sclerosis"[MeSH Terms] OR  "multiple sclerosis"[Title/Abstract] ) |

**STable 2. Summary and thematic interpretation of keyword clusters identified by VOSviewer**

| **Cluster** | **No. of keywords** | **Representative keywords** | **Proposed thematic label** | **Thematic interpretation** |
| --- | --- | --- | --- | --- |
| Cluster 1 (red) | 133 | adverse childhood experiences; childhood trauma; abuse; maltreatment; stress; depression; obesity; inflammation; risk factors; chronic pain; posttraumatic stress disorder; women | *Adverse childhood experiences and multidimensional chronic health consequences* | This cluster centers on adverse childhood experiences and their associations with psychological stress, depression, obesity, inflammation, chronic pain, posttraumatic stress disorder, and other long-term health consequences. It reflects the broad disease burden and potential biopsychosocial pathways associated with childhood adversity. |
| Cluster 2 (green) | 91 | childhood adversity; child; cohort studies; longitudinal studies; epidemiology; risk factors; cardiovascular disease; body mass; health behavior; smoking; mortality; pregnancy | *Life-course epidemiology and cardiometabolic risk* | This cluster mainly represents population-based and longitudinal research examining how childhood adversity influences health across the life course. Particular attention is given to behavioral risk factors, obesity, cardiovascular disease, mortality, pregnancy-related outcomes, and other cardiometabolic consequences. |
| Cluster 3 (blue) | 73 | child abuse; child sexual abuse; emotional abuse; emotional neglect; anxiety disorder; bipolar disorder; cognition; eating disorder; chronic fatigue syndrome; comorbidity; somatization; mental health | *Childhood maltreatment, mental disorders, and somatic comorbidity* | This cluster focuses on specific forms of childhood maltreatment and their relationships with psychiatric disorders, cognitive impairment, emotional and behavioral problems, chronic fatigue, somatization, and psychiatric-somatic comorbidity. |

Note: Representative keywords were selected according to occurrence frequency, total link strength, network position, and thematic relevance. Cluster labels and thematic interpretations were assigned by the authors based on the semantic characteristics of the representative keywords.

**STable 3. Summary and thematic interpretation of keyword clusters identified by CiteSpace**

| **Cluster ID** | **Size** | **Silhouette** | **Mean year** | **LLR-derived label** | **Representative keywords** | **Thematic interpretation** |
| --- | --- | --- | --- | --- | --- | --- |
| #0 | 106 | 0.632 | 2017 | *Adverse childhood experience* | adverse childhood experience; early life adversity; childhood maltreatment; childhood trauma; mood disorder; chronic inflammation; breast cancer; later life | Broad chronic health consequences and biological pathways of childhood adversity. This cluster reflects multidimensional long-term outcomes involving psychiatric, inflammatory, oncological, and later-life health domains. |
| #1 | 97 | 0.711 | 2017 | *Childhood maltreatment* | childhood maltreatment; adverse childhood experience; childhood trauma; opioid use; internalizing symptoms; trauma-informed care; national survey; later life | Childhood maltreatment, psychological consequences, and trauma-informed care. The cluster emphasizes internalizing symptoms, substance-related outcomes, and trauma-informed health services. |
| #2 | 84 | 0.627 | 2019 | *Chronic fatigue syndrome* | chronic fatigue syndrome; functional somatic syndrome; early life stress; irritable bowel syndrome; visceral hypersensitivity; brain development; childhood adversity | Functional somatic syndromes and neurobiological effects of early-life stress. Major topics include chronic fatigue, gastrointestinal symptoms, visceral sensitivity, and brain-related mechanisms. |
| #3 | 68 | 0.564 | 2020 | *Food addiction* | food addiction; uncontrolled eating; eating disorder; bariatric surgery candidate; weight stigma; child diet; young women; posttraumatic stress disorder symptoms | Disordered eating, food addiction, and obesity-related care. The cluster represents trauma-related eating behaviors, weight stigma, bariatric populations, and associated psychosocial factors. |
| #4 | 67 | 0.645 | 2019 | *Later life* | later life; cardiovascular health; cardiovascular diseases; blood pressure; ambulatory blood pressure; early life stress; social support; posttraumatic stress disorder | Life-course cardiovascular health and later-life risk. The cluster examines blood-pressure and cardiovascular outcomes and the possible influence of psychosocial support across the life course. |
| #5 | 64 | 0.659 | 2019 | *Oncology patient* | oncology patient; receiving chemotherapy; breast cancer; breast cancer patient; childhood maltreatment; sexual abuse; childhood trauma; irritable bowel syndrome | Childhood adversity in oncology and breast cancer populations. This cluster highlights cancer-related experiences, particularly among breast cancer patients and individuals receiving chemotherapy. |
| #6 | 27 | 0.736 | 2020 | *Genetic risk* | genetic risk; genotype-environment effect; pregnant women; maternity care; waist circumference; weight gain; reproductive strategy; early-life adversity | Genetic, reproductive, and anthropometric pathways. The cluster concerns gene-environment interplay, pregnancy and maternity care, body composition, and weight-related outcomes. |

**Note:** Cluster labels were generated using the log-likelihood ratio (LLR) method in CiteSpace. Representative keywords were selected from the LSI- and LLR-derived terms according to thematic relevance. Thematic interpretations were assigned by the authors based on the semantic characteristics of the cluster labels and representative terms. Cluster size indicates the number of nodes included in each cluster.
